# Hospital Administration

**Published:** 1886-10-02

**Authors:** 


### Hospital Administration.

Communications for this column should be addressed, "Administrator," care of the Editor of THE HOSPITAL.

Explanatory. THERE can be no doubt that much of the criticism which sometimes finds its way into the correspondents' column of the daily press is due to an absence of precise knowledge and to the want of a judicial agency to which inquiries can be addressed by members of the public, with the certainty that the information supplied will be accurate, fair, and impartial. The Hospitals Association, Norfolk House, Norfolk Street, Strand, W.C., the President of which is Sir Andrew Clark, Bart., M.D., F.R.S., and the Chairman of the Council Major Ross, M.P., Chairman of the Middlesex Hospital, has been quietly working to bring about a system of co-operation amongst hospital managers throughout the country, which will make its office a centre of information to which all may apply with confidence, when any need of precise information upon any matter relating to the administration and management of hospitals and asylums may arise. The hospitals generally, and the provincial institutions especially, have recognised the utility of the work which the Hospitals Association has undertaken in the interests of all the institutions and the public, and the statistics already collected with the aid of the authorities of these institutions are of interest and value. It is proposed to utilise this column for the purpose of making public such portions of the information collected from time to time by the Hospitals Association as may seem to be best calculated to interest and help those who are engaged in hospitals and asylums, or who support or use the hospitals. It is hoped that this effort will be cordially welcomed, and that all who have it in their power to assist in the work will readily avail themselves of the facilities for mutual help and instruction here afforded.

Mr. George Sturge's efforts. Few, perhaps, take a more genuine interest in much that concerns the welfare of hospitals, certainly few have more liberally supported these institutions, than Mr. George Sturge of Sydenham Hill. This gentleman has, within the last few years, distributed amongst several of the hospitals very large sums of money; and it is within our knowledge that he desires to do his utmost to promote the efficiency of the management, especially on those points which relate to questions in which he has taken a lifelong interest. We wish further to say that we are aware that his efforts in connection with the consumption of alcohol in hospitals have been neither unfruitful nor devoid of benefit to the best interests of the patients.

Mr. Sturge's question answered. There is, however, a time for all things, as there should certainly be a limit to the criticisms of an enthusiast upon any given point of administration which he regards as of paramount importance. Certain letters which Mr. Sturge has recently addressed to the *British Medical Journal*, and especially one which is published in last Saturday's issue, however well intentioned, cannot commend themselves to the judgment of those who recognise the difficulties with which hospital managers have to contend, and the exceptionally trying character of the life and duties which devolve upon the resident staff, and especially upon the medical and nursing staff in a hospital. It is perfectly true that some people, and probably the majority of people, living ordinary lives, and being under forty years of age, are often better when they refrain altogether from the habitual use of alcohol. But the atmosphere and surroundings of the hospital resident, especially where residence has extended over a period of years, often necessitates the taking of a small daily portion of alcohol in the case of certain individuals so circumstanced, if ordinary health is to be maintained. Besides, restriction upon the individual liberty of the employed by any employer is an act not to be tolerated or supported in a free country like England. When, therefore, Mr. Sturge asks, "Are the managers of our hospitals justified in using charity funds, given for the use of the sick and suffering, to supply alcoholic drink to the hospital staff?" the emphatic answer must be "Yes, most certainly they are, because the health of the staff is of paramount importance to the proper performance of the hospital work."

Mr. Sturge's figures. Mr. Sturge refers to two hospitals, and states that the value of alcohol consumed in them by the staff amounted to about £400 and £218 respectively in a given year. It must be evident that such a statement is of no practical value without further information as to the number of the staff in each hospital; for if he has in his mind a hospital like the London, the £400 would give a sum little in excess of £1 per diem to provide alcohol for the whole staff, and would not probably represent the cost of a glass of bitter beer per head per diem. But Mr. Sturge implies that he is obliged to be indefinite, and to make general statements of this character, because the accounts rendered by the hospitals are not precise enough, and it is therefore impossible to state the exact amount expended upon alcohol per annum: (*a*) for the staff, and (*b*) for the patients. This statement might reasonably lead the uninitiated to suppose that the hospitals were unwilling to supply the necessary information and to court the fullest investigation into the facts. This would be a very erroneous conclusion. To formulate such a belief is to do a serious injustice to the hospital managers throughout the country; for we have found, by addressing an inquiry to each of the hospitals, that within a week a reply has been sent, stating fully all the facts, so far as they are in the possession of the managers themselves.

These facts, which we shall publish as an act of justice to the hospitals, tend to prove that Mr. Sturge, however well-intentioned, cannot, rightly, continue his alcohol crusade against the hospitals, seeing that of their own initiative during the last twenty years they have steadily given the most careful consideration to this question, with the result, that the amount of alcohol now consumed by staff and patients, as compared with twenty years ago, is so reduced as to render it impossible for any reasonable man to believe in either a want of care or an excess of consumption.

